# Response to “A commentary on ‘Leveraging diverse cell-death patterns to predict the prognosis and drug sensitivity of triple-negative breast cancer patients after surgery’”

**DOI:** 10.1097/JS9.0000000000002298

**Published:** 2025-02-05

**Authors:** Jindong Xie, Xinpei Deng, Anli Yang, Shaoquan Zheng, Yuhui Tang, Yi Xie, Junsheng Zhang, Hailin Tang, Wenkuan Chen, Yutian Zou, Xiaoming Xie

**Affiliations:** a State Key Laboratory of Oncology in South China, Guangdong Provincial Clinical Research Center for Cancer, Department of Surgical Oncology, Sun Yat-sen University Cancer Center, Guangzhou, China; b The First Affiliated Hospital, Sun Yat-sen University, Guangzhou, China

*Dear Editor,*

We express our gratitude for the insightful commentary offered by Li *et al*^[1]^ regarding our study, “Leveraging diverse cell-death patterns to predict the prognosis and drug sensitivity of triple-negative breast cancer patients after surgery”^[2]^. Their insights significantly contribute to the continuous development and enhancement of this study, and we would like to provide some perspectives to these comments.

Programmed cell death (PCD) might ensue when damage is extensive and repair mechanisms fail to provide protection or support recovery, or it may be a result of intrinsic self-regulatory processes. Different PCD patterns play a crucial role in tumor progression and can act as prognostic markers and predictors of drug sensitivity in triple-negative breast cancer (TNBC), one of the most aggressive breast cancer subtypes. We developed an innovative metric, the cell death index (CDI), to forecast the effectiveness of therapeutic interventions as well as prognostic outcomes in TNBC patients. Our research indicates that the PCD-related gene signature serves as a valuable prognostic tool for postoperative TNBC patients, significantly improving the assessment of clinical outcomes.

In our original study, we acknowledged several limitations. For instance, we recognized that the patients were retrospectively recruited from public cohorts, which might inherently cause bias. Second, compared to the well-known Oncotype DX (21-gene) and MammaPrint (70-gene) assays which have been validated in multiple clinical trials and are widely recommended in guidelines with high reliability and reproducibility, our CDI model lacks prospective, randomized, and controlled trials to validate the decision-making effectiveness, and might face high detection cost as well as technical threshold in practical operation. Nevertheless, our CDI model has greater potential for personalization and scalability in predicting the efficacy of specific treatments, such as immunotherapy or targeted therapy. Furthermore, the biological functions of certain PCD genes in our model have not been investigated in TNBC cells, indicating a critical area for future experimental research. Regarding these limitations, we have retrospectively collected a certain amount of TNBC patients’ paraffin-embedded tissues from our center, and we plan to conduct RT-qPCR and RNA-seq analyses to obtain the expression levels of incorporated PCD genes to validate the reliability of our CDI model. Moreover, we are actively engaging with several research institutions and clinical hospitals to obtain external multi-center validations of our CDI model.

We also highlighted that as researchers continue to develop new technologies, our study did not incorporate emerging multi-omics methodologies (such as spatial transcriptome analysis, proteomics, and metabolomics) to confirm our findings, as proposed in oncology research^[3-5]^. Spatial transcriptome analysis might identify the cell types and CDI patterns in different regions of tumor interior and periphery, shedding light on the interaction network among tumor cells, immune cells and stromal cells, while also identifying the mechanisms affecting tumor immune escape. Besides, spatial transcriptome analysis could recognize the spatial distribution of different clonal populations and explore its relationship with treatment resistance. Although limited proteomics data are available, the expression levels and modification states of proteins, as direct functional executors, provide more accurate reflections on the dynamic characteristics of disease progression. Integrating multi-omics data into functional modules (such as pathways or networks) through hierarchical analysis, or incorporating protein functional features as additional model predictors, might enhance the predictive ability and biological interpretability of existing model. Moreover, with the finding of other novel cell death types such as disulfidptosis^[6]^, ammonium-induced death^[7]^, and triaptosis^[8]^, the integration of these novel cell death types into the existing model and their subsequent development warrant careful consideration. We believe that future studies could help to address these limitations, and we look forward to further research in this area to confirm and expand upon our findings.

In conclusion, we believe that our study offers novel insights that substantiate the application of PCD in the treatment of TNBC. We appreciate the authors’ commentary and believe that further research is needed to explore its potential applications. Furthermore, we are confident that our study, being the first to incorporate multiple cell death patterns in cancer, offers a promising method for forecasting the prognosis and drug sensitivity of TNBC patients after surgery. We eagerly anticipate continued dialogue and research in this field, aiming to advance our comprehension and utilization of PCD model in the field of translational oncology.

## Data Availability

Not applicable.
